# Expression of *AtLEC2* and *AtIPTs* promotes embryogenic callus formation and shoot regeneration in tobacco

**DOI:** 10.1186/s12870-019-1907-7

**Published:** 2019-07-15

**Authors:** Ke Li, Juan Wang, Chuanliang Liu, Changsheng Li, Jingjing Qiu, Chuanzhi Zhao, Han Xia, Changle Ma, Xingjun Wang, Pengcheng Li

**Affiliations:** 1 College of Life Sciences, Shandong University, Qingdao, 266237 People’s Republic of China; 2 Biotechnology Research Center, Shandong Academy of Agricultural Sciences, Shandong Provincial Key Laboratory of Crop Genetic Improvement, Ecology and Physiology, Jinan, 250100 People’s Republic of China; 3 College of Life Sciences, Shandong Normal University, Jinan, 250014 People’s Republic of China; 4 Institute of Cotton Research of Chinese Academy of Agricultural Sciences, Anyang, 455000 People’s Republic of China

**Keywords:** GR, Dexamethasone, *AtLEC2*, *AtIPT7*, *AtIPT9*, Embryogenic callus, Shoot regeneration, Transcriptome analysis

## Abstract

**Background:**

LEAFY COTYLEDON 2 (LEC2) acts throughout embryo morphogenesis and maturation phase to maintain embryogenic identity. Our previous study stated that *Arabidopsis thaliana LEC2* (*AtLEC2*) driven by glucocorticoid receptor-dexamethasone (GR-DEX) inducible system (*AtLEC2-GR*) triggers embryogenic callus formation in tobacco (*Nicotiana tabacum*).

**Results:**

In this study, the adenosine phosphate isopentenyltransferase genes *AtIPT3*, *AtIPT7* and the tRNA isopentenyltransferase gene *AtIPT9* were overexpressed in the *AtLEC2-GR* transgenic background. In the *AtIPT7-OE AtLEC2-GR* and *AtIPT9-OE AtLEC2-GR* seedlings, high-quality embryogenic callus was obtained under the DEX condition, and the shoot regeneration efficiency was 2 to 3.5 folds higher than *AtLEC2-GR* alone on hormone free medium without DEX. Transcriptome analyses showed that up-regulated *BBM*, *L1L*, *ABI3*, and *FUS3* might function during embryogenic callus formation. However, at the shoot regeneration stage, *BBM*, *L1L*, *ABI3*, and *FUS3* were down-regulated and Type-B *ARRs* were up-regulated, which might contribute to the increased shoot regeneration rate.

**Conclusions:**

A novel system for inducing shoot regeneration in tobacco has been developed using the GR-DEX system. Induced expression of *AtLEC2* triggers embryogenic callus formation and overexpression of *AtIPT7* or *AtIPT9* improves shoot regeneration without exogenous cytokinin.

**Electronic supplementary material:**

The online version of this article (10.1186/s12870-019-1907-7) contains supplementary material, which is available to authorized users.

## Background

Genetic improvement through the transgenic technology has been widely used for many crops, however, the transformation and regeneration of some crops were proved to be difficult and genotype dependent [1]. Establishment of a more efficient shoot regeneration system is of great significance for crop genetic improvement. Plant regeneration can be accomplished through somatic embryogenesis and organogenesis [2]. Traditional protocol for shoot regeneration is achieved by two steps: embryogenic callus initiation on the auxin-rich medium; shoot meristem formation on cytokinin-rich medium [3].

Transcriptional factors are known to play significant roles in plant cell differentiation and dedifferentiation. Overexpression of a number of transcriptional factor genes can improve somatic embryogenesis and enhance plant regeneration, such as *LEAFY COTYLEDON 1* (*LEC1*) [4], *LEAFY COTYLEDON 2* (*LEC2*) [5, 6], *WUSCHEL* (*WUS*) [7], *BABY BOOM* (*BBM*) [8] and *AGAMOUS-LIKE 15* (*AGL15*) [9]. *LEC1*/*LEC1-LIKE* (*L1L*) with three B3 domain protein genes *ABSCISIC ACID* (*ABA*)*-INSENSITIVE3* (*ABI3*), *FUSCUA3*(*FUS3*) and *LEC2* is referred as *LAFL* network (*LEC1*/*L1L*, *ABI3*, *FUS3* and *LEC2*) [10]. This network functions redundantly throughout the early embryo developmental process, embryo maturation and dormancy in a dose-dependent manner [11]. Ectopic expression of *FUS3* and *ABI3* enhances accumulation of embryo traits but without somatic embryogenesis [12, 13]. *YUCCA4* (*YUC4*) encodes a protein that catalyzes the rate-limiting step in IAA biosynthesis [14]. LEC2 can interact with FUS3 and bound to the *YUC4* promoter [15]. Ectopic expression of *LEC2* rapidly activates *YUC2* and *YUC4* [15]. These observations suggest that the LEC2 induced embryogenic competence is tightly linked with the auxin. Furthermore, inactivation of gibberellic acids (GAs) biosynthesis enzymes or reduction of active GAs also enhances the embryogenic competence of tissue [16]. LEC2 directly activates the expression of *AGL15* [9, 17]. AGL15 and FUS3 have been reported to decrease GAs contents through negatively regulation of *gibberellin 20-oxidase1* (*GA20ox1*), *GA3ox1* and *GA3ox2* GAs biosynthesis enzyme encoding genes [13, 18]*.* Therefore, the embryogenic competence of LEC2 is also associated with GAs activity. In addition, the expression of *LAFL* genes is regulated at both chromatin level and transcriptional level. Two AINTEGUMENTA-LIKE (AIL) family transcriptional factors, BBM and PLETHORA2 (PLT2), may directly activate *LAFL* transcription [19]. VIVIPAROUS1/ABI3-LIKE 1 (VAL1) and chromatin remodeler PICKLE-RELATED 2 (PKR2) inhibit the *LAFL* gene expression [20–22], CURLY LEAF (CLF), the member of Polycomb Repressive Complex 2 (PRC2) inhibits the *LEC2* transcription [23, 24].

LEC2 can trigger vegetative to embryogenic transition, however, plant regeneration could not occur from embryogenic callus constitutively expressing LEC2 [5, 6]. The GR is a vertebrate steroid hormone receptor. It has been reported that the GR-DEX system is a good induction system in plants because DEX, a strong synthetic glucocorticoid, itself does not cause any pleiotropic effects in plants [25–27]. Under no DEX condition, transcription factor (TF)-GR as a cytoplasmic complex with heat shock protein (HSP90), and the binding of DEX to GR leads to the dissociation of HSP90, and causes nucleus localization of TF-GR [25]. GR, as a transcription factor, could also activate transcription of the glucocorticoid response elements (GREs) containing promoters, in the presence of a glucocorticoid [26–28]. Indeed, our previous study has reported that the tobacco plants with induced expression of *AtLEC2* by the GR-DEX system (*AtLEC2-GR*) display an obvious somatic embryogenesis phenotype and shoots could be generated from the embryogenic callus under no DEX condition [6].

Cytokinins control cell division and cell differentiation, as well as shoot growth and apical dominance. It is well-known that the appropriate ratio of cytokinin and auxin promotes shoot formation [3]. Isopentenyltransferases (IPTs) catalyzes the rate-limiting step of the cytokinin biosynthesis [29]. IPT was initially found in *Dictyostelium discoideum* and *Agrobacterium tumefaciens*, it has been proved to convert adenosine-5′-monophosphate (AMP) and dimethylallyl pyrophosphate (DMAPP) into isopentenyladenine riboside 5′ -monophosphate (iPMP) [30, 31]. Constitutive expression of *IPT* from the Ti-plasmid of *A. tumefaciens* significantly elevates the cytokinin levels in transgenic plants and results in excessive cytokinin abnormal phenotype [31, 32]. However, inducible expression of *IPT* from the *Agrobacterium tumefaciens* by the GR-DEX system can induce suitably elevated cytokinin levels and shoot formation [27]. In Arabidopsis, there are two types of isopentenyltransferases (IPTs): one type (AtIPT1 and AtIPT3 to AtIPT8) catalyzes adenosine phosphates (ATP/ADP or AMP) to react with DMAPP, another type (AtIPT2 and AtIPT9) catalyzes the isopentenylation of tRNA [33]. *AtIPTs* show diverse temporal and spatial expression patterns [34–36]. Overexpression of *AtIPT4* enhanced shoot regeneration efficiency independent of external cytokinins [33]. Furthermore, the *AtIPT8* gain-of-function mutant remarkably increases iPMP and Isopentenyl adenosine (iPA) levels, improves embryogenic callus formation and shoot regeneration [37]. The cytokinin response requires the participation of the hormone-dependent Cytokinin Receptor 1 (CRE1) and hormone-independent CYTOKININ-INDEPENDENT 1 (CKI1), the His-containing phosphotransfer factors (*Arabidopsis thaliana* histidine phosphotransfer proteins, AHPs), His kinases (HKs) and Arabidopsis response regulators (ARRs) [38]. These factors are involved in transferring of phosphoryl groups between their conserved His and Asp, to control gene expression and global physiological response [38–42]. Recent studies reveal that type-B *ARRs* (*ARR1*, *ARR10* and *ARR12*) can activate the expression of *WUSCHEL* (*WUS*), to maintain the shoot apical meristem and axillary meristem. Furthermore, these proteins can negatively regulate *YUCCAs* (*YUC1* and *YUC4*) to inhibit auxin accumulation [43–45]. However, shoot regeneration of the *yuc1 yuc4* double mutant is dramatically decreased, suggesting that auxin is also indispensable in the determination of cell fate [43].

High regeneration and transformation efficiency are crucial for gene engineering-based crop genetic modification. Many studies have attempted to increase somatic embryogenesis and shoot regeneration rate through ectopic expressing key genes, such as *LEC2*, *SOMATIC EMBRYOGENESIS RECEPTOR-LIKE KINASE* (*SERKs*), *WUS* and *BBM* [6, 7, 46–50]. Lowe et al. (2016) recently reported that co-expression of maize *BBM* and *WUS2* genes significantly increases transformation frequency in commercial maize inbred lines, sorghum, sugarcane, and indica rice. Here, we described a simple and efficient regeneration system that combines the use of *AtLEC2-GR* and *AtIPT7* or *AtIPT9* overexpression. The system relied on the DEX-inducible expression of *AtLEC2* to trigger embryogenic callus formation and overexpression of *AtIPT7* or *AtIPT9* to promote shoot regeneration. This regeneration system enabled embryogenic callus formation under the DEX condition and shoot regeneration after the removal of DEX.

## Results

### Exogenous cytokinin promotes shoot regeneration from embryogenic callus

**Fig. 1:**
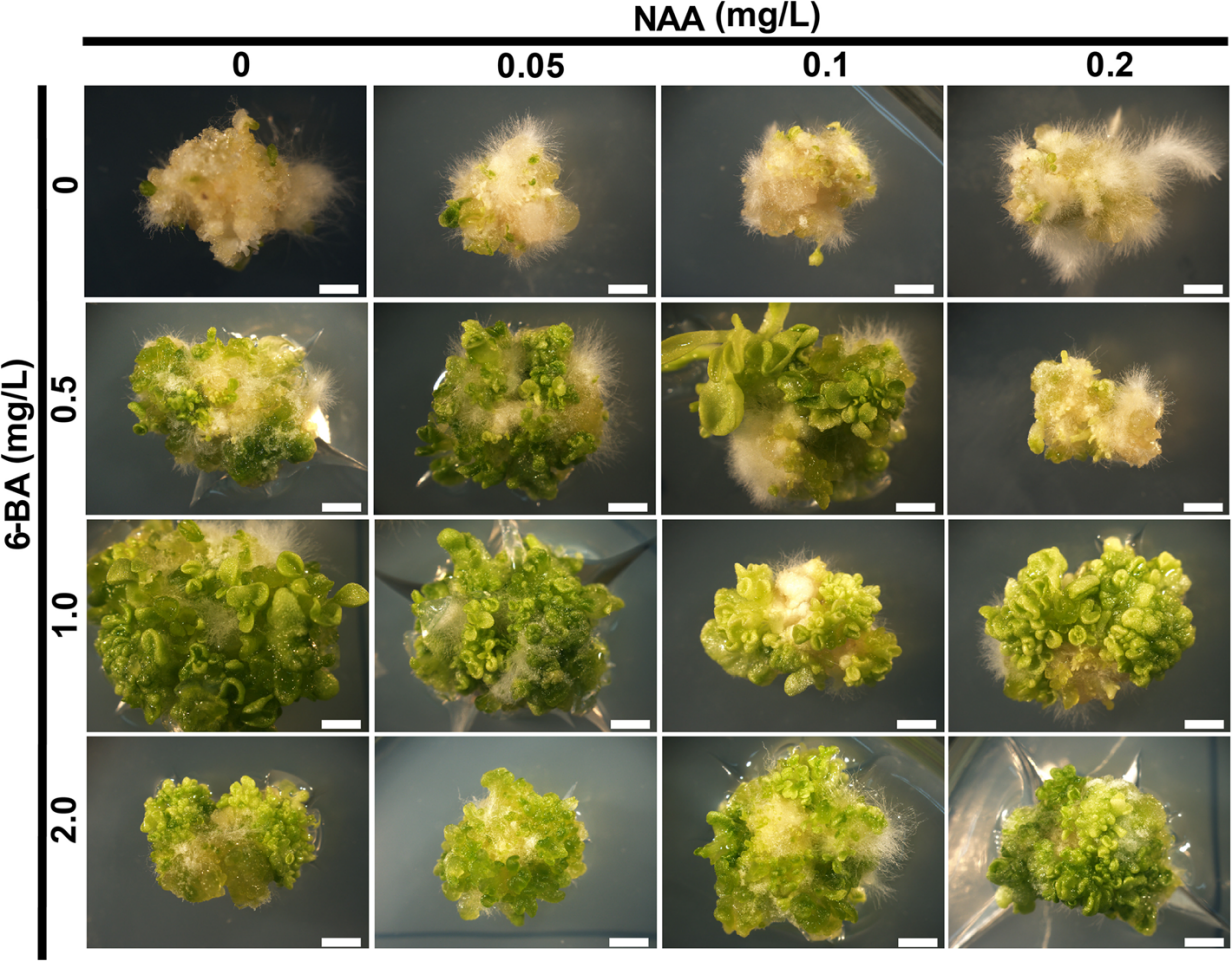
Shoot regeneration from embryogenic callus derived from *AtLEC2-GR* lines with exogenous application of NAA and 6-BA**.** Embryogenic callus derived from *AtLEC2-GR* transgenic seedlings grown on MS medium with (0, 0.05, 0.1, and 0.2 mg/L) NAA and (0, 0.5, 1.0, and 2.0 mg/L) 6-BA. Embryogenic callus grown on MS medium with 0 mg/L NAA and 0 mg/L 6-BA were as control. Three independent experiments were performed, each experiment contains 5 replicates. Scale bars = 2 mm

Induced expression of *AtLEC2* under the DEX condition can generate embryogenic callus on the shoot apical meristem (SAM) in transgenic tobacco [6]. Shoots could be generated from the embryogenic callus on hormone-free MS medium (no DEX), but with a low regeneration efficiency [6]. In order to increase the shoot regeneration rate, exogenous 1-naphthylacetic acid (NAA) and 6-Benzylaminopurine (6-BA) were applied. We found that 6-BA could significantly promote shoot regeneration. The number of regenerated shoots was increased 1.2 to 2 folds when a low concentration of NAA was applied when compared with the hormone-free MS medium (Fig. 1 and Table 1). Remarkably, a 2.9 to 8.1 folds increase in the number of regenerated shoots was observed when the embryogenic callus treated with a low concentration of 6-BA compared with the hormone free MS medium (Fig. 1 and Table 1). We further found that the application of 0.05 mg/L NAA together with 1.0 mg/L 6-BA led to a maximum shoots regeneration rate (Fig. 1 and Table 1). These results demonstrated that exogenous cytokinin could significantly promote the shoot regeneration efficiency of embryogenic callus from the *AtLEC2-GR* transgenic line.

**Table 1:** Shoot regeneration numbers on MS medium with exogenous hormones NAA and 6-BA**.** Values are mean ± standard errors form 15 replicates. Three asterisks indicate statistically significant differences from the control (NAA contents 0 mg/l and 6-BA contents 0 mg/l) numbers (^***^P < 0.001, Student’s *t*-test)

NAA contents (mg/l)	6-BA contents (mg/l)	Shoot regeneration numbers
0	0	7.7 ± 1.1
0	0.5	52.0 ± 2.5 ***
0	1	65.7 ± 4.0 ***
0	2	22.0 ± 2.5 ***
0.05	0	17.5 ± 2.3 ***
0.05	0.5	62.0 ± 4.3 ***
0.05	1	69.5 ± 4.2 ***
0.05	2	30.9 ± 1.9 ***
0.1	0	13.7 ± 2.2 ***
0.1	0.5	40.1 ± 2.6 ***
0.1	1	49.5 ± 2.7 ***
0.1	2	42.4 ± 1.8 ***
0.2	0	10.9 ± 2.6 ***
0.2	0.5	20.6 ± 1.8 ***
0.2	1	40.3 ± 2.1 ***
0.2	2	34.1 ± 3.0 ***

### Generation of transgenic plants and phenotypic analysis

The prominent effect of exogenous cytokinin treatment encouraged us to promote the shoot regeneration efficiency from the *AtLEC2-GR* embryogenic callus through increasing endogenous cytokinin concentration. *IPTs* are key genes in the cytokinin biosynthesis pathway. *AtIPT3*, *AtIPT7* and *AtIPT9* were proved mainly expressed in phloem tissues or proliferating tissue of the growing seedling [36]. *AtIPT3*、*AtIPT7* and *AtIPT9* overexpressed vectors driven by the CaMV *35S* promoter were constructed and then transformed into the *AtLEC2-GR* transgenic tobacco (*AtIPT3-OE AtLEC2-GR*, *AtIPT7-OE AtLEC2-GR* and *AtIPT9-OE AtLEC2-GR*, respectively) respectively by the *Agrobacterium-*mediated leaf disc transformation [51]. Totally, 13 *AtIPT3-OE AtLEC2-GR*, 15 *AtIPT7-OE AtLEC2-GR* and 18 *AtIPT9-OE AtLEC2-GR* independent transgenic lines were generated, respectively. As shown in Additional file 1: Figure S3 the *AtLEC2*, *AtIPT3*, *AtIPT7* and *AtIPT9* fragments were amplified by PCR using specific primers in the indicated transgenic lines. The result suggested that *AtIPT3*, *AtIPT7* and *AtIPT9* had been stably integrated into the genomes of *AtLEC2-GR* transgenic tobacco. In the subsequent experiment, three independent *AtIPTs-OE AtLEC2-GR* transgenic lines of each construct were selected for further studies.

Homozygous *AtIPTs-OE AtLEC2-GR* transgenic seeds were germinated on MS medium with or without the DEX induction. The comparisons of the *AtIPTs-OE AtLEC2-GR* lines with wild type and *AtLEC2-GR* lines were carried out. Under no DEX condition, no remarkable phenotype difference was observed among the lines of *AtIPTs-OE AtLEC2-GR*, *AtLEC2-GR* and wild type plants (Additional file 1: Figure S1a-S1e). The *AtIPTs-OE AtLEC2-GR* lines had slightly longer hypocotyls than the *AtLEC2-GR* and wild type seedlings at 10 DAG (days after germination), on the contrary, the primary root length of *AtIPTs-OE AtLEC2-GR* was 22.3, 34.6 and 16.9% shorter than the control seedlings, respectively (Additional file 1: Figure S1f). The *AtIPTs-OE AtLEC2-GR* plants grew well in the soil and more axillary buds could develop into shoots during the vegetative growth phase (Additional file 1: Figure S2a-S2d). In addition, we found that overexpression of *AtIPTs* dramatically increased the floral number at the reproductive stage (Additional file 1: Figure S2e-S2 h).

**Fig. 2:**
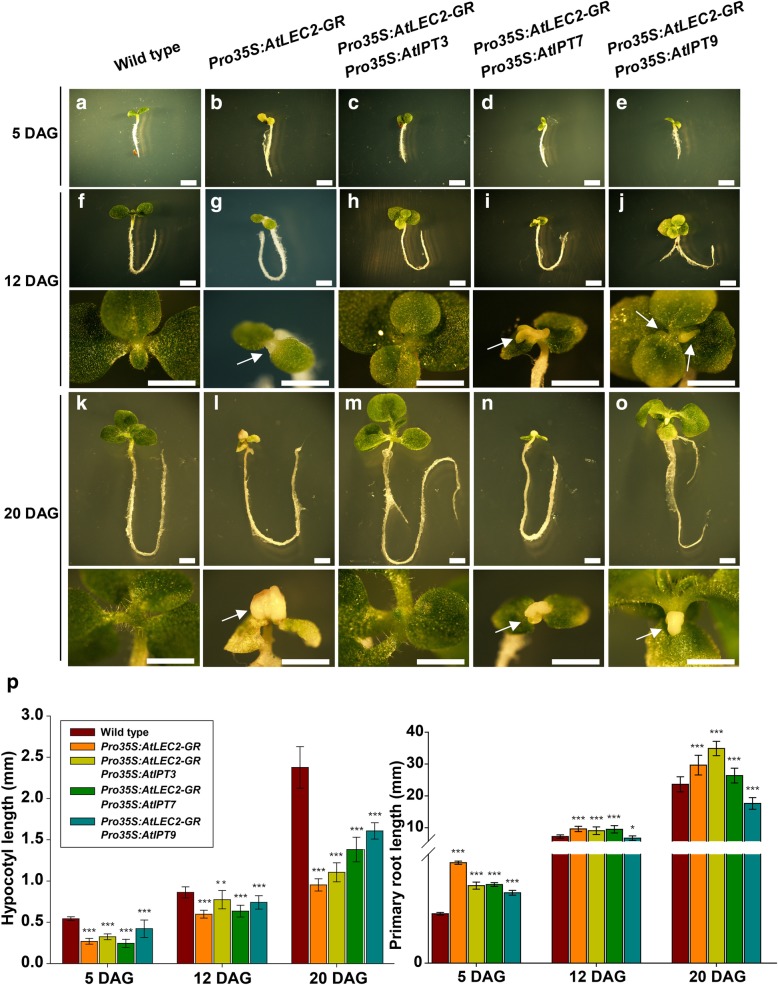
Seedlings of wild type and different transgenic lines grown on 20 μM DEX containing medium**. a**-**e** The phenotype of 5 DAG seedlings under 20 μM DEX induction. Scale bars = 2 mm. **f**-**j** The upper part shows the 12 DAG seedlings under 20 μM DEX induction. Scale bars = 2 mm; the lower part shows the enlarged images. Scale bars = 6 mm. **k**-**o** The upper part shows the 20 DAG seedlings under 20 μM DEX induction. Scale bars = 2 mm; the lower part shows the enlarged images. Scale bars = 6 mm. **p** Measurement of the hypocotyl (Left) and primary root (Right) lengths of seedlings. Values are mean ± standard errors form 20 replicates. Single, double, and three asterisks indicate statistically significant differences from the wild type (at the corresponding DAG: 5 DAG, 12 DAG, and 20 DAG, respectively) (^*^P < 0.05, ^**^P < 0.01, and ^***^P < 0.001, Student’s *t*-test). The pictures were captured using line 5 of *AtIPT3-OE AtLEC2-GR* transgenic lines, line 9 of *AtIPT7-OE AtLEC2-GR* transgenic lines and line 8 of *AtIPT9-OE AtLEC2-GR* transgenic lines. White arrows point to embryogenic callus

When grown on MS medium with 20 μM DEX, we could not observe embryogenic callus formation on the SAM in all 13 *AtIPT3-OE AtLEC2-GR* lines (Fig. 2h, m, Additional file 1: Figure S5), while embryogenic callus appeared on the SAM of *AtIPT7-OE AtLEC2-GR* lines at 8–12 DAG (Fig. 2i, n, Additional file 1: Figure S5). Interestingly, compared to the *AtLEC2-GR* and *AtIPT7-OE AtLEC2-GR* plants, *AtIPT9-OE AtLEC2-GR* plants produced two pieces of embryogenic callus on the peripheral zone of SAM at 12 DAG and the normal growth of leaves was not affected (Fig. 2j, o, Additional file 1: Figure S5). The *AtLEC2-GR* plants had obviously shorter hypocotyls than wild type, but the *AtIPTs-OE AtLEC2-GR* plant hypocotyls were longer compared with the *AtLEC2-GR* background at 20 DAG (Fig. 2p left). *AtIPT3-OE AtLEC2-GR* and *AtIPT7-OE AtLEC2-GR* had longer primary roots than wild type, and *AtIPT3-OE AtLEC2-GR* had the longest primary root (Fig. 2p right). *AtIPT9-OE AtLEC2-GR* had the shortest primary root at 20 DAG (Fig. 2n, o, Additional file 1: Figure S5). Same as *AtLEC2-GR*, the *AtIPT7-OE AtLEC2-GR* seedlings had fleshy and unexpanded cotyledons, and the growth of seedling was ceased (Fig. 2l, n, Additional file 1: Figure S5). We also tested the phenotypes of all transgenic seedlings grown on MS medium with 50 μM DEX. Unexpectedly, no embryogenic callus was formed from 13 *AtIPT3-OE AtLEC2-GR* lines (Additional file 1: Figure S4b). Meanwhile, for the *AtIPT7-OE AtLEC2-GR* and *AtIPT9-OE AtLEC2-GR* transgenic lines, the embryogenic callus formation was the same as the 20 μM DEX condition (Additional file 1: Figure S4c-S4d). We determined the *trans*-zeatin (*t*Z) -type cytokinin contents in the 20 DAG *AtIPTs-OE AtLEC2-GR*, *AtLEC2-GR* and wild type seedlings grown on MS medium with 20 μM DEX, but no significant change was observed (data not shown).

### Overexpression of *AtIPTs* promotes shoot regeneration efficiency

**Fig. 3:**
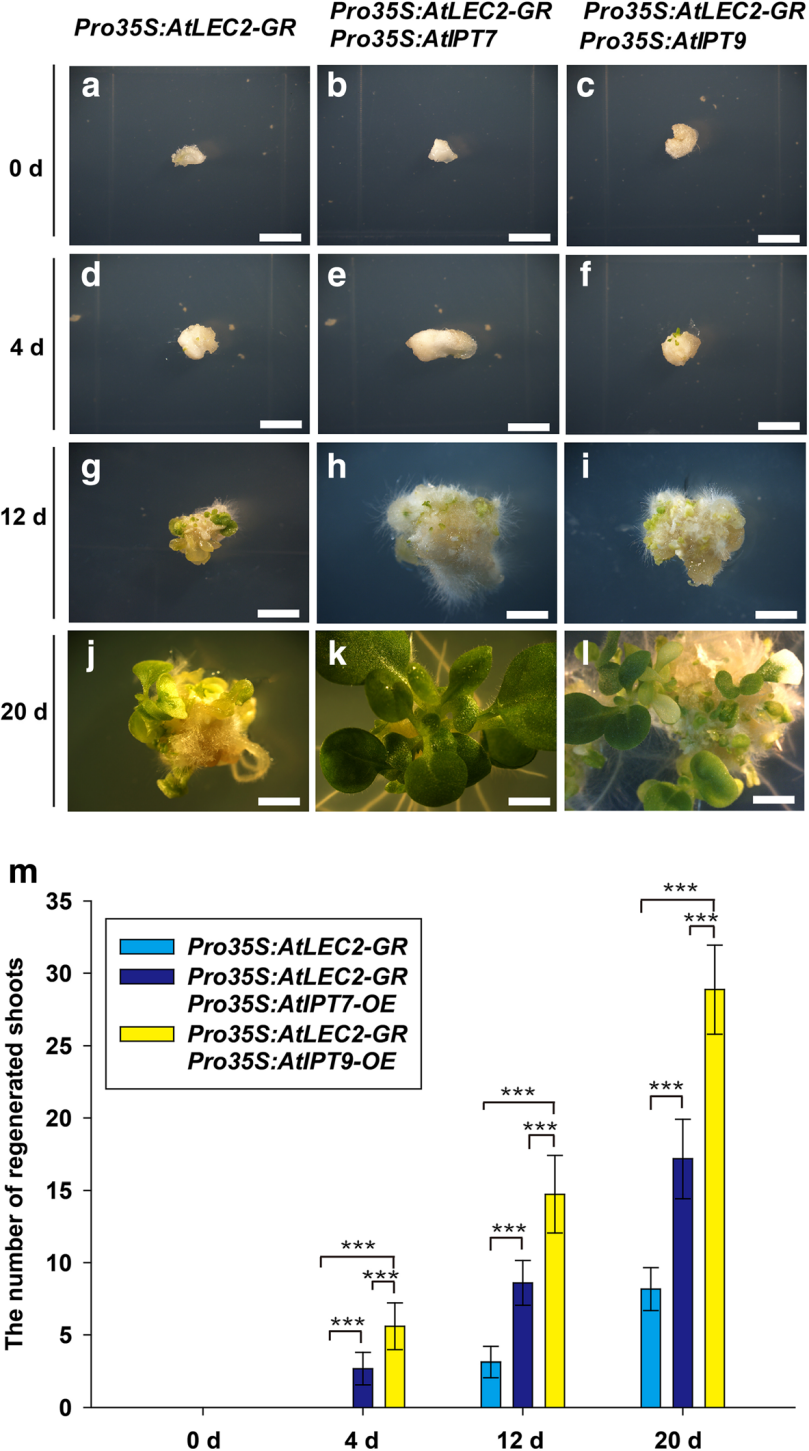
Shoot regeneration from callus derived from different transgenic lines**. a**-**c** Embryogenic callus separated from SAM of 20 DAG seedlings under 20 μM DEX induction. Scale bars = 3 mm. **d**-**f** 4 d after embryogenic callus on hormone-free MS medium (no DEX). Scale bars = 3 mm. **g**-**i** 12 d after embryogenic callus on hormone-free MS medium (no DEX). Scale bars = 3 mm. **j**-**l** 20 d after embryogenic callus on hormone-free MS medium (no DEX). Scale bars = 3 mm. **m** Statistics of shoot regeneration number of embryogenic callus on hormone-free MS medium (no DEX). Cyan indicated embryogenic callus derived from three independent *AtLEC2-GR* transgenic lines (each line contains 20 replicates). Dark blue indicated embryogenic callus derived from three independent *AtIPT7-OE AtLEC2-GR* transgenic lines (line 9, 10 and 11, and each line contains 20 replicates). Yellow indicated embryogenic callus derived from three independent *AtIPT9-OE AtLEC2-GR* transgenic lines (line 8, 9 and 12, and each line contains 20 replicates). Values are mean ± standard errors form 60 replicates. Three asterisks indicate statistically significant differences (^***^P < 0.001, Student’s *t*-test). d, days after transferred to hormone-free MS medium

Embryogenic callus derived from the 20 DAG seedlings of *AtIPT7-OE AtLEC2-GR*, *AtIPT9-OE AtLEC2-GR* and *AtLEC2-GR* under the 20 μM DEX induction were cultured on the hormone-free MS medium without DEX (Fig. 3a-c). Two days later, the proliferation of the embryogenic callus and many somatic embryo-like structures on the callus could be observed. Some of the somatic embryos could develop into shoots (Fig. 3). After 4 d, 3–5 shoots developed from *AtIPT7-OE AtLEC2-GR* and *AtIPT9-OE AtLEC2-GR* somatic embryos, but not from *AtLEC2-GR* callus (Fig. 3d-f, m). After 12 d, 3–14 shoots regenerated from the embryogenic callus derived from *AtLEC2-GR*, *AtIPT7-OE AtLEC2-GR* and *AtIPT9-OE AtLEC2-GR* transgenic plants (Fig. 3g-i, m). On an average, 8 shoots could generate from one piece of *AtLEC2-GR* embryogenic callus and 16 shoots from the *AtIPT7-OE AtLEC2-GR* embryogenic callus. Significantly, 28 shoots could generate from one embryogenic callus of *AtIPT9-OE AtLEC2-GR* (Fig. 3j-l, m). Shoots on the surface of embryogenic callus were able to develop into healthy plants (Additional file 1: Figure S6).

### Transcriptome analysis of different transgenic plants

**Fig. 4:**
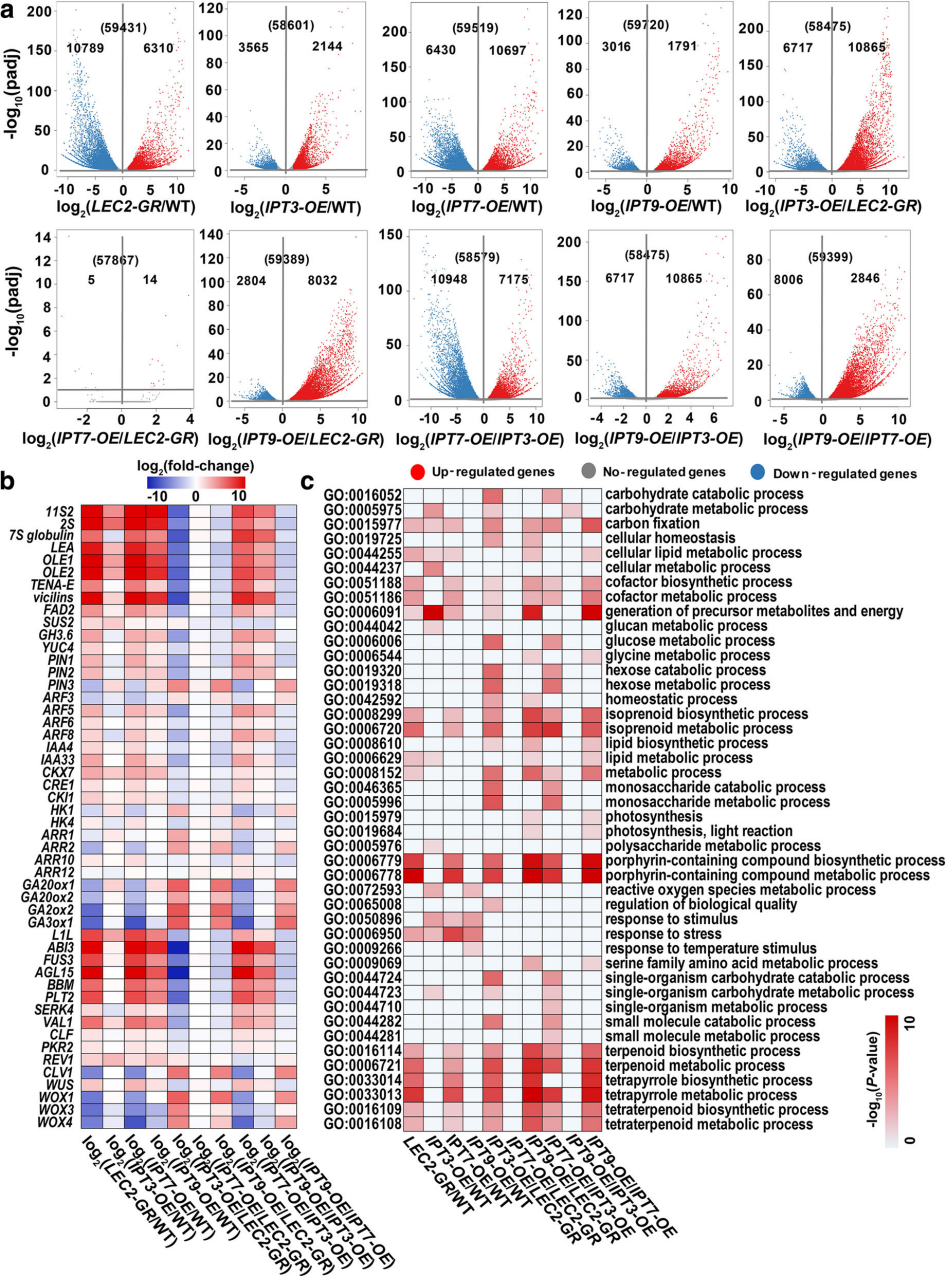
Gene expression profiling of wild type and different transgenic lines under 20 μM DEX induction**. a** Volcano plots of fold changes. Log_2_ of fold-change and -log_10_ of the padj are present on the axis. Red, blue and gray dots represent significantly up-, down-regulated and no-regulated genes (**|**Log_2_ (fold-change)**|** > 1, padj < 0.1). **b** Interested genes analysis use the log_2_-transformed fold-change values. Red, green and black indicate increase, decrease and no difference of expression levels, respectively. **c** GO functional categories of genes with transcriptionally up- and down-regulated levels for the indicated comparisons. The color in each cell indicates -log_10_ (*P*-values) of the GO enrichment according to the scale shown. Identification of significantly (*P*-values < 0.05) enriched GO categories. padj, adjusted *P*-values

To understand the mechanism by which *AtIPTs* differentially influenced embryogenic callus formation, 20 DAG seedlings of wild type, *AtLEC2-GR*, and *AtIPTs-OE AtLEC2-GR* grown under the 20 μM DEX induction were used for gene expression profiling. To distinguish genes regulated by ectopic expression of *AtLEC2* and *AtIPTs*, we carried out a comparative analysis of genes expression between these different lines (Fig. 4). When *AtLEC2-GR* was compared with the wild type (*LEC2-GR*/WT) plants, 6310 genes were up-regulated and 10,789 genes were down-regulated (Fig. 4a). Gene Ontology (GO) analysis of differentially expressed genes revealed enrichment in isoprenoid metabolic process (GO:0006720), porphyrin-containing compound biosynthetic and metabolic process (GO:0006779 and GO:0006778) (Fig. 4c). Embryogenic storage protein genes including *11S2*, *2S*, *7S globulin* and *vicilins*, and late embryogenesis abundant (LEA) protein genes were significantly upregulated. The expression of *SUCROSE SYNTHASE 2* (*SUS2*) and *FATTY ACID DESATURASE 2* (*FAD2*) genes were upregulated (Fig. 4b). In addition, genes implicated in the regulation of embryo development, somatic embryogenesis and seed maturation, including *LAFL* network genes, *AGL15*, and AIL family transcription factor genes (*BBM* and *PLT2*) were significantly up-regulated in the *AtLEC2-GR* seedlings (Fig. 4b). The expression of many key regulators involved in auxin, abscisic acid, cytokinin and gibberellin acid metabolism and signaling were changed by ectopic *AtLEC2* expression. For example, *YUC4*, encoding a flavin monooxygenase enzymes involved in auxin biosynthesis, was induced (Fig. 4b). *AtLEC2* also activated PIN-FORMED (PIN) auxin efflux facilitators (*PIN1*, *PIN2* and *PIN3*) and auxin response factors (ARFs) (*ARF5*, *ARF6* and *ARF8*) (Fig. 4a, b). In contrast, the genes encoding GA20ox1, GA20ox2 and GA3ox1, which catalyzed the later steps of gibberellic acid biosynthesis, were down-regulated (Fig. 4b). Three epigenetic repressor genes *CURLY LEAF* (*CLF*), *VP1*/*ABI3-LIKE 1* (*VAL1*) and CHD3-type chromatin-remodeling factor *PICKLE RELATED 2* (*PKR2*), were up-regulated (Fig. 4b).

Overexpressing *AtIPT3*, *AtIPT7* and *AtIPT9* genes led to different phenotypes as described above. We also investigated the change of gene expression in three *AtIPTs-OE AtLEC2-GR* transgenic seedlings. In *AtIPT7-OE AtLEC2-GR*, 10697 genes were up-regulated and 6430 genes were down-regulated compared with wild type (*IPT7-OE*/WT) (Fig. 4a). GO enrichment was observed in the porphyrin-containing compound biosynthetic and metabolic process (GO:0006779 and GO:0006778) and response to stress (GO:0006950) and so on. (Fig. 4c). The *AtIPT7-OE AtLEC2-GR* seedlings exhibited a similar changing tendency compared with *AtLEC2-GR*. Consistent with the phenotype, only 14 up-regulated genes and 5 down-regulated genes were detected between *AtIPT7-OE AtLEC2-GR* and *AtLEC2-GR* lines (*IPT7-OE*/*LEC2-GR*) (Fig. 4a). In *AtIPT9-OE AtLEC2-GR*, a total of 1791 up-regulated genes and 3016 down-regulated genes were identified when *AtIPT9-OE AtLEC2-GR* compared with wild type plant (*IPT9-OE*/WT). GO terms of response to stimulus (GO:0050896), response to stress (GO:0006950) and so on, were enriched in the differentially expressed genes (Fig. 4c). Eight thousand thirty-two genes were up-regulated and 2804 genes were down-regulated when compared between *AtIPT9-OE AtLEC2-GR* and *AtLEC2-GR* (*IPT9-OE*/*LEC2-GR*) plants (Fig. 4a). Generation of precursor metabolites and energy (GO:0006091), isoprenoid biosynthetic and metabolic process (GO:0008299 and GO:0006720) and so on, were enriched in the differentially expressed genes (Fig. 4c). Gene expression in *AtIPT9-OE AtLEC2-GR* was compared with *AtIPT7-OE AtLEC2-GR* (*IPT9-OE*/*IPT7-OE*), 2846 genes were up-regulated and 8006 genes were down-regulated (Fig. 4a). Carbon fixation (GO:0015977), cofactor metabolic process (GO:0051186) and generation of precursor metabolites and energy (GO:0006091) and so on, were enriched in the differentially expressed genes (Fig. 4c). In *AtIPT3-OE AtLEC2-GR* seedlings, 2144 genes were up-regulated and 3565 genes were down-regulated when compared with the wild type (*IPT3-OE*/WT). GO analysis of differentially expressed genes revealed enrichment in carbohydrate metabolic process (GO:0005975), generation of precursor metabolites and energy (GO:0006091) and response to stimulus (GO:0050896) (Fig. 4c). Ten thousand eight hundred sixty-five and 6717 genes were up- and down-regulated when compared with *AtLEC2-GR* (*IPT3-OE*/*LEC2-GR*) (Fig. 4a). Carbohydrate catabolic process (GO:0016052), carbon fixation (GO:0015977) and cellular homeostasis (GO:0019725) and so on, were enriched in the differentially expressed genes (Fig. 4c). However, in the *AtIPT3-OE AtLEC2-GR* seedlings, embryo storage proteins and *LEA* proteins encoding genes, key somatic embryogenesis and seed maturation regulator genes were up-regulated. Furthermore, *PIN1*, *PIN2*, *GRETCHEN HAGEN3.6* (*GH3.6*), *YUC4*, *ARF5*, *ARF8* and *GA20ox2* were expressed oppositely when compared with *AtLEC2-GR* and two other *AtIPTs-OE AtLEC2-GR* plants (Fig. 4b). These results indicated that the SAM microenvironment for embryogenic callus formation was differentially affected by ectopic expression of these genes. Finally, 7175 genes were up-regulated and 10,948 genes were down-regulated in *AtIPT7-OE AtLEC2-GR* when compared to *AtIPT3-OE AtLEC2-GR (IPT7-OE*/*IPT3-OE*). Carbohydrate catabolic process (GO:0016052), carbon fixation (GO:0015977) and hexose metabolic process (GO:0019318) and so on, were enriched in the differentially expressed genes (Fig. 4c). Ten thousand eight hundred sixty-five genes were up-regulated and 6717 genes were down-regulated in *AtIPT9-OE AtLEC2-GR* when compared to *AtIPT3-OE AtLEC2-GR* (*IPT9-OE*/*IPT3-OE*) (Fig. 4a). Carbohydrate metabolic process (GO:0005975) was enriched in the differentially expressed genes (Fig. 4c). All results demonstrated that the down-regulation of embryo-specific protein genes, auxin synthesis and signaling genes, and the up-regulation of active GA biosynthesis enzyme genes negatively affected the formation of embryogenic callus in the *AtIPT3-OE AtLEC2-GR* seedlings (Fig. 4b).

**Fig. 5:**
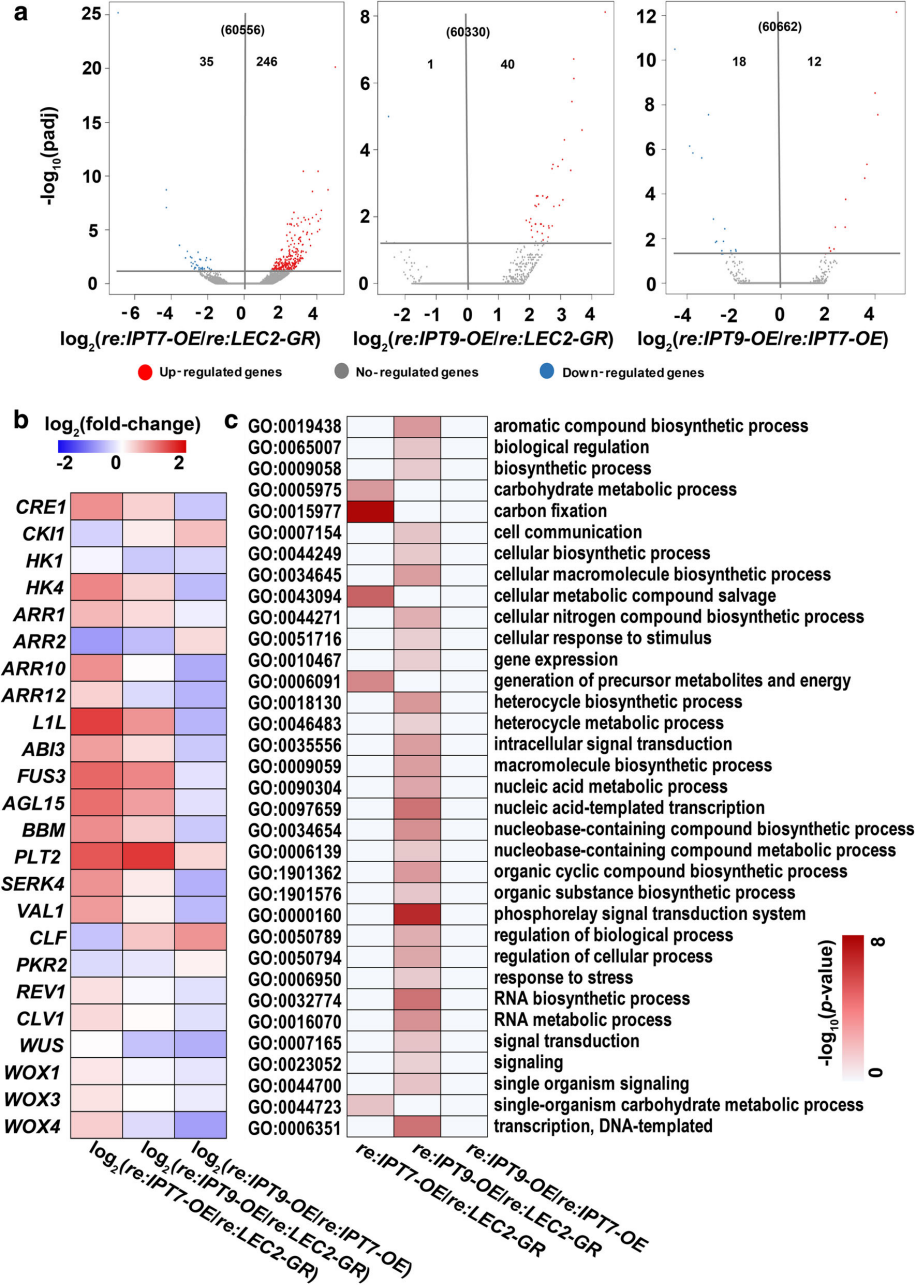
Gene expression profiling of callus derived from different transgenic lines after 20 days regeneration**. a** Volcano plots of fold changes. Log_2_ of fold-change and -log_10_ of the padj are present on the axis. Red, blue and gray dots represent significantly up-, down-regulated and no-regulated genes (**|**Log_2_ (fold-change) **|** > 1, padj < 0.1). **b** Interested genes analysis use the log_2_-transformed fold-change values. Red, green and black indicate increase, decrease and no difference of expression levels. **c** GO functional categories of genes with transcriptionally up- and down-regulated levels for the indicated comparisons. The color in each cell indicates -log_10_ (*P*-values) of the GO enrichment according to the scale shown. Identification of significantly (*P*-values < 0.05) enriched GO categories

To illustrate the mechanisms by which AtIPT7 and AtIPT9 promoted shoot regeneration efficiency, 20 days callus derived from *AtIPT7-OE AtLEC2-GR*, *AtIPT9-OE AtLEC2-GR* and *AtLEC2-GR* seedlings after regeneration on hormone-free MS medium (*re: AtIPT7-OE AtLEC2-GR*, *re: AtIPT9-OE AtLEC2-GR* and *re: AtLEC2-GR*, respectively) were used for gene expression profiling. Totally, 246 genes were up-regulated and 35 genes were down-regulated when compared *re: AtIPT7-OE AtLEC2-GR* with *re: AtLEC2-GR* (*re: IPT7-OE/re: LEC2-GR*, Fig. 5a). GO analysis of differentially expressed genes revealed enrichment in carbon fixation (GO:0015977) and cellular metabolic compound salvage (GO:0043094) and so on (Fig. 5c). In *re: AtIPT7-OE AtLEC2-GR*, cytokinin signaling genes (*CRE1*, *HK4*, *ARR1*, *ARR10* and *ARR12*), *LAFL* genes, *AGL15*, *BBM*, *PLT2*, *REVOLUTA* (*REV*), *CLAVATA 1* (*CLV1*), *WUSCHEL RELATED HOMEOBOX 1* (*WOX1*), *WOX3* and *WOX4* were activated (Fig. 5b). *ARR2* and two epigenetic regulators (*CLF* and *PKR2*) were down-regulated (Fig. 5b). In addition, up-regulated genes in *re: AtIPT7-OE AtLEC2-GR* had a similar expression pattern in *re: AtIPT9-OE AtLEC2-GR*, except for *ARR12*, *WUS* and *WOX4* (Fig. 5b). When compared *re: AtIPT9-OE AtLEC2-GR* with *re: AtLEC2-GR* (*re: IPT9-OE*/*re: LEC2-GR*), 40 genes were up-regulated and only one gene was down-regulated (Fig. 5a). GO analysis of differentially expressed genes revealed enrichments in nucleic acid-templated transcription (GO:0097659), phosphorelay signal transduction system (GO:0000160), RNA biosynthetic process (GO:0032774) and so on (Fig. 5c). The expression of *CKI1*, *ARR1* and *PLT2* in *re: AtIPT9-OE AtLEC2-GR* line were up-regulated when compared to *re: AtLEC2-GR*. This result was consistent with the gene expression profiling in 20 DAG seedlings (Fig. 4b). The expression of 12 genes was up-regulated and 18 genes were down-regulated when compared *re: AtIPT9-OE AtLEC2-GR* with *re: AtIPT7-OE AtLEC2-GR* (*re: IPT9-OE*/*re: IPT7-OE*, Fig. 5a). Except for *CKI1*, *ARR2*, *PLT2* and *CLF*, all genes listed in Fig. 5b were down-regulated.

## Discussion

Biotechnology has become a useful means for crop genetic improvement through overexpressing or silencing the key genes. In most cases, the genes were driven by the 35S promoter or the ubiquitin promoter. However, a more flexible gene expression system is essential, especially for genes that would lead to abnormal phenotype when constitutively expressing. The GR-DEX system has been considered a desired induction system which is simple and efficient, and DEX could function within four hours through direct addition to the medium or spray on the explants [26]. Our previous research showed that induced expression of *AtLEC2* could promote vegetative to embryogenic transition and formed embryogenic callus on the SAM of the transgenic tobacco [6]. This phenotype enabled us to obtain embryogenic callus through application of DEX. An indispensable requirement for shoot regeneration and growth is the removal of *AtLEC2* expression on DEX free medium (Fig. 3 and Additional file 1: Figure S6) [41]. Similarly, constitutive expression of *WUS* or *BBM* trigger somatic embryogenesis [7, 8]. There are other ways to terminate the expression of the transgenes. For example, Lowe et al. (2016) used a drought-inducible promoter to drive CRE expression and terminate the gene expression by the removal of the *Bbm* and *Wus2* sequences between the loxP sites [47].

In this study, multiple endogenous cytokinins could be affected by overexpressing the *AtIPT* genes and resulted in high efficiency of shoot regeneration. Plant transformation has been blocked by several bottlenecks, including genotype and specific culture medium dependence [1]. The expression of *AtIPTs* conferred cell ability to increase endogenous cytokinin levels and to regulate downstream target genes expression, which might play a more precise role than direct application of exogenous cytokinins and could make the regeneration process convenient, cost-saving and without selecting exogenous cytokinin species and concentrations. In previous researches, overexpressed *IPT* from *A. tumefaciens* usually triggers excessive cytokinin abnormal phenotype [31, 32]. Here, we found that overexpression of *AtIPT7* and *AtIPT9* enhanced shoots regeneration from embryogenic callus, especially the *AtIPT9* (Fig. 3j-l). Although we did not find an increased accumulation of the *t*Z-type cytokinin in three *AtIPTs-OE AtLEC2-GR* seedlings, other cytokinin derivatives might be increased [52]. The phenotype of *AtIPTs-OE AtLEC2-GR* plants with increased numbers of lateral branch also implies an enhanced cytokinin accumulation (Additional file 1: Figure S2a-S2 h). Sun et al. (2003) reported that overexpression of *AtIPT8* only causes higher levels accumulation of iPMP and iP (isopentenyladenosine), rather than *t*Z or other cytokinins. Because no callus was obtained in *AtIPT3-OE AtLEC2-GR*, we are unable to confirm if *AtIPT3* has the same effect on the shoot regeneration (Fig. 2m, Additional file 1: Figure S5). In our study, transgenic tobacco overexpressing *AtIPTs* didn’t exhibit excessive cytokinin phenotype. The plants grew well and have more axillary branches (Additional file 1: Figure S2a-S2d). In addition, overexpression of *AtIPTs* could increase the floral number at the reproductive stage (Additional file 1: Figure S2e-S2 h) and delay plant senescence (data not shown).

Transcription profiling analysis demonstrated that ectopic expression of *AtLEC2* in tobacco activated many genes encoding embryo proteins and embryo storage proteins, consistent with its regulation roles during embryo maturation [17]. Up-regulated expression of *FAD2* and *SUS2* suggested that the ectopic *LEC2* activity might increase the fatty acid and sucrose accumulation. Overexpression of *AtIPTs* in the *AtLEC2* background differently affected embryogenic callus formation (Fig. 2a-o). Firstly, we found embryogenic callus was not formed in the *AtIPT3-OE AtLEC2-GR* seedlings (Fig. 2m). It has been reported that *BBM*, *PLT2* and *LAFL*, *AGL15* induce embryogenesis in a dose-dependent manner [17, 19]. Consistent with this phenotype, gene expression of embryo development regulators, *BBM*, *PLT2*, *LAFL* and *AGL15*, were all significantly down-regulated in the *AtIPT3-OE AtLEC2-GR* seedlings (Fig. 4b). According to the above results, we proposed that AtIPT3 or its product might inhibit the expression of *BBM*, *PLT2*, *LAFL* and *AGL15* or their downstream target genes, but the detailed mechanism remains exclusive. Down-regulation of embryo storage proteins and embryogenesis abundant proteins genes also testified our point of view (Fig. 4b). In addition, the down-regulation of auxin biosynthesis genes (*GH3.6* and *YUC4*), auxin polar transport genes (*PIN1* and *PIN2*) and auxin signaling genes (*ARF5*, *ARF8* and *IAA33*) in the *AtIPT3-OE AtLEC2-GR* seedlings implicated that the microenvironment on SAM for callus formation was affected (Fig. 4b). In contrast, in *AtIPT7-OE AtLEC2-GR* and *AtIPT9-OE AtLEC2-GR* seedlings, the genes encoding embryo development regulators, *BBM*, *PLT2*, *LAFL* and *AGL15*, embryo traits, auxin metabolism and signal transduction genes have a similar expression tendency compared with the *AtLEC2-GR* seedlings (Fig. 4b). It has been reported that biologically active GAs played negative roles in embryogenic callus formation [6, 53]. Here, we found that genes active GAs synthesis, like *GA20ox2* and *GA3ox1*, were down-regulated in *AtLEC2-GR*, *AtIPT7-OE AtLEC2-GR* and *AtIPT9-OE AtLEC2-GR* when compared with the wild type plants. In contrast, *GA20ox1*, and *GA20ox2* were up-regulated in *AtIPT3-OE AtLEC2-GR* when compared with the wild type plants (Fig. 4b). Further analysis revealed that *GA20ox1*, *GA20ox2* and *GA3ox1* were up-regulated in *AtIPT3-OE AtLEC2-GR* when compared with *AtLEC2-GR* (Fig. 4b). Our results suggested that the up-regulation of GA biosynthesis enzyme genes might be another reason that caused the inhibition of embryogenic callus formation in the *AtIPT3-OE AtLEC2-GR* seedlings.

Previous studies have demonstrated that overexpression of *LEC1*, *BBM* and *AGL15* promotes shoot regeneration [4, 8, 9]. Here, we found that the expressions of *BBM*, *LAFL* and *AGL15* genes were significantly up-regulated in the *AtIPT7-OE AtLEC2-GR* and *AtIPT9-OE AtLEC2-GR* plants (Fig. 4b). This elevated expression level of *BBM*, *LAFL* and *AGL15* in transgenic tobacco could be a key factor leading to the high rate of shoot regeneration; however, the excess expression could have an opposite effect. This explanation was consistent with our previous results that 10 μM DEX induction led to a higher regeneration rate than 30 and 50 μM DEX induction [6]. Callus formation was initiated on the DEX containing medium, while further development of the somatic embryo and shoot formation required removal of DEX induction. We found that *BBM*, *LAFL* and *AGL15* had a lower expression level in *AtIPT9-OE AtLEC2-GR* than *AtIPT7-OE AtLEC2-GR* (Fig. 4b). Even though no shoots were generated on embryogenic callus in this stage, the cell fate for shoot regeneration had been determined. Furthermore, they displayed a similar expression pattern in *re: AtIPT9-OE AtLEC2-GR* compared to *re: AtIPT7-OE AtLEC2-GR* (Fig. 5b). The up-regulation of two negative regulators, *CLF* and *PRK2*, consistent with the down-regulation of *L1L* and *FUS3* in *re: AtIPT9-OE AtLEC2-GR* when compared with *re: AtIPT7-OE AtLEC2-GR* (Fig. 5b)*.* Taken together, it is very likely that the expression patterns of *BBM*, *LAFL* and *AGL15* are the major reasons for the embryogenic callus formation and promoted shoot regeneration, however, excess expression level of these genes might inhibit shoot formation.

Here, we found cytokinin receptors *CRE1*, *HK4* and three type-B *ARRs* (*ARR1*, *ARR10* and *ARR12*) in *AtIPT7-OE AtLEC2-GR* and *re: AtIPT7-OE AtLEC2-GR* were activated when compared with *AtLEC2-GR* and *re: AtLEC2-GR* (Fig. 4b and Fig. 5b). In *AtIPT7-OE AtLEC2-GR*, *CRE1*, *CKI1*, *HK4* and *ARR10* were down-regulated whereas *HK1*, *ARR1* and *ARR12* were up-regulated when compared with *AtLEC2-GR* (Fig. 4b). In *re: AtIPT7-OE AtLEC2-GR*, we found that *CRE1*, *HK4*, *ARR1*, *ARR10* and *ARR12* were up-regulated (Fig. 5b). Meanwhile, *CRE1*, *CKI1*, *HK4*, *ARR1* and *ARR10* were up-regulated in *re: AtIPT9-OE AtLEC2-GR* (Fig. 5b). Above results might explain that the improved shoot regeneration efficiency when overexpression of *AtIPT7* and *AtIPT9* might be due to enhanced cytokinin response. *HK4*, *ARR10* and *ARR12* might contribute to the shoot regeneration improvement in *re: AtIPT7-OE AtLEC2-GR*. However, *HK4* and *ARR1* played the function in *re: AtIPT9-OE AtLEC2-GR*. The recent study revealed that B-type *ARRs* directly activated the expression of *WUS* and repressed the *YUCs* transcription [43–45]. *WUS* has been shown to participate in the stem cell specification and somatic embryogenesis. We found that *WUS* was up-regulated in *AtIPT7-OE AtLEC2-GR*, but down-regulated in *AtIPT9-OE AtLEC2-GR* when compared with *AtLEC2-GR* (Fig. 4b). *WUS* was up-regulated in *re: AtIPT7-OE AtLEC2-GR* and down-regulated in *re: AtIPT9-OE AtLEC2-GR* (Fig. 5b). These results indicated that there might have other factors responsive to cytokinin derivatives produced by AtIPT9 and promoted shoot regeneration. Down-regulation of *CLV1* in *re: AtIPT9-OE AtLEC2-GR* than in *re: AtIPT7-OE AtLEC2-GR* further enriched *WUS* expression range and promoted the regeneration of additional shoots [54]. Recently, Zhang et al. (2017) demonstrated that *WOX1*, *WOX2*, *WOX3* and *WOX5* redundantly maintain the balance between the cytokinin and auxin pathways and function in the initiation of the stem cell. However, WUS is dispensable for this process in embryogenic shoot stem cell initiation. In addition, terminal branches and leaf-like structures could still form in Arabidopsis *wus* mutants [44]. All these reports further supported the existence of additional regulators redundantly in SAM initiation.

## Conclusion

In this work, we established a simple and efficient regeneration system by co-expression of *AtLEC2* and *AtIPT7* or *AtIPT9*. The induced expression of *AtLEC2* triggers embryogenic callus formation and overexpression of *AtIPT7* or *AtIPT9* improves shoot regeneration without exogenous cytokinins. This strategy might be useful for crop genetic improvement in the future.

### Accession number

The accession numbers of the genes used in this article are as follows: *AtLEC2* (*AT1G28300*), *AtIPT3* (*AT3G63110*), *AtIPT7* (*AT3G23630*), and *AtIPT9* (*AT5G20040*).

## Methods

### Plant materials and growth conditions

Seeds of tobacco (*Nicotiana tabacum* cv. SR1) were stored in our laboratory. Seeds of *Arabidopsis thaliana* ecotype Columbia-0 (Col-0) were obtained from the Arabidopsis Biological Resource Center (ABRC, Ohio State University, Columbus, OH, USA). Seeds were surface-sterilized in 75% ethanol for 30 s and then in 10% (v/v) H_2_O_2_ for 10 min, and washed four times in sterile distilled water. Arabidopsis seeds were sown on solid 1/2 Murashige and Skoog (MS) medium containing 1% sugar. After 2 days at 4 °C in darkness, seeds were.

transferred to white light with 100 μmol m^− 2^ s^− 1^ for 16 h and darkness for 8 h daily at 22 °C. Tobacco seeds were sown on solid MS medium containing 4% sugar and grown at white light with 100 μmol m^− 2^ s^− 1^ for 16 h and darkness for 8 h daily at 25 °C.

### Plasmid constructs and gene transformation

The coding sequences (CDS) of *AtIPT3*, *AtIPT7* and *AtIPT9* were amplified from *A. thaliana* using specific primers (Additional file 1: Table S1). The PCR amplification was performed with the following parameters: 95 °C for 5 min; 36 cycles of 95 °C for 30 s, 58 °C for 1 min, 72 °C for 1 min; and 72 °C for 10 min. The *AtIPT3*, *AtIPT7* and *AtIPT9* CDS fragments were confirmed by sequencing and clone into *pCAMBIA2300.* Pro35S: *AtIPT3*, *Pro35S: AtIPT7 and Pro35S: AtIPT9* constructs were transferred into *Agrobacterium tumefaciens* (LBA4404) and transformed into *Pro35S:AtLEC2-GR* transgenic tobacco (*Nicotiana tabacum* cv. SR1) using leaf discs method [55]. The *AtIPT3-OE AtLEC2-GR*, *AtIPT7-OE AtLEC2-GR* and *AtIPT9-OE AtLEC2-GR* transgenic tobacco were selected on MS medium containing 50 mg/L kanamycin. Genomic DNA was isolated from the T2 transgenic seedlings. The transgenic lines were further confirmed by PCR amplification using specific primers of *AtLEC2*, *AtIPT3*, *AtIPT7* and *AtIPT9* (Additional file 1: Table S1). PCR amplification was performed with the following parameters: 95 °C for 5 min; 36 cycles of 95 °C for 30 s, 58 °C for 1 min, 72 °C for 1 min; and 72 °C for 10 min.

### DEX induction and shoot regeneration from transgenic seedlings

DEX (25 mM) stock solution was dissolved in absolute ethanol and added to MS medium at a final concentration of 20 μM. Homozygotic transgenic seeds were germinated on MS medium containing DEX for 20 days to obtain embryogenic callus and used for imaging or in vitro shoot regeneration. Callus from *AtLEC2-GR*, *AtIPT7-OE AtLEC2-GR* and *AtIPT9-OE AtLEC2-GR* was transferred to MS medium without DEX and exogenous hormone for shoot regeneration.

### Microscopy and photograph

Images were captured with OLYMPUS SZX16 microscope and Canon EOS 500D camera. The lengths of hypocotyl and primary root were measured by the measuring tool in OLYMPUS SZX16 microscope. Adobe Illustrator and Photoshop were used for final image arrangement and annotations.

### Transcriptome sequencing and data analysis

To gain insight into the mechanism by which *AtIPTs* influenced embryogenic callus formation, total RNA was isolated from wild type, *AtLEC2-GR*, *AtIPT3-OE AtLEC2-GR*, *AtIPT7-OE AtLEC2-GR* and *AtIPT9-OE AtLEC2-GR* seedlings grown for 20 d on MS medium containing 20 μM DEX for the RNA-sequencing (RNA-seq) experiment. To illustrate the mechanisms that *AtIPT7* and *AtIPT9* promote shoot regeneration efficiency, we isolated total RNA from *AtLEC2-GR*, *AtIPT7-OE AtLEC2-GR* and *AtIPT9-OE AtLEC2-GR* callus after grown on hormone-free MS medium for the RNA-seq experiment. RNA-seq was performed by BGISEQ-500 platform (Shenzhen, China). The data were provided by The Beijing Genomics Institute (BGI). The significantly regulated genes were screened according to Log_2_ of fold-change and -log_10_ of the p*adj* (adjusted *p*-value) (**|**Log_2_ (fold-change) **|** > 1, padj < 0.1). Identification of significantly (*p*-value < 0.05) enriched GO categories was done using a web-based tool and database for GO analysis (http://www.geneontology.org/).

## Additional files

Additional file 1: **Figure S1.** Phenotype of seedlings grown on hormone free MS medium without DEX. **Figure S2.** Phenotype of mature plants grown in soil. **Figure S3.** PCR amplified the *AtLEC2*, *AtIPT3*, *AtIPT7* and *AtIPT9* fragments. **Figure S4.** Seedlings grown on 50 μM DEX condition. **Figure S5.** Seedlings grown on 20 μM DEX containing medium. **Figure S6.** Shoot regeneration from the callus. **Table S1.** Primers used in the study. (DOCX 1921 kb)

## Data Availability

The data sets supporting the results of this article are included within the article and its additional files.

## Abbreviations

6-BA: N^6^-benzyladenine
ABI3: ABSCISIC ACID (ABA)-INSENSITIVE3
AGL15: AGAMOUS-LIKE 15
AHPs: *Arabidopsis thaliana* histidine phosphotransfer proteins
AMP: Adenosine-5′-monophosphate
ARFs: AUXIN RESPONSE FACTORs
ARRs: Arabidopsis response regulators
BBM: BABY BOOM
CKI1: CYTOKININ-INDEPENDENT 1
CLF: CURLY LEAF
CLV1: CLAVATA 1
CRE1: cytokinin receptor 1
DEX: Dexamethasone
DMAPP: dimethylallyl pyrophosphate
FAD2: FATTY ACID DESATURASE 2
FUS3: FUSCUA3
GA20ox1: Gibberellin 20-oxidase1
GAs: gibberellic acids
GH3.6: GRETCHEN HAGEN3.6
GR: Glucocorticoid receptor
GREs: glucocorticoid response elements
HKs: His kinases
HSP90: Heat shock protein
iPA: Isopentenyl adenosine
iPMP: Isopentenyladenine riboside 5′ –monophosphate
IPT: Isopentenyltransferase
L1L: LEC1/LEC1-LIKE
LAFL: (LEC1/L1L, ABI3, FUS3 and LEC2)
LEA: late embryogenesis abundant
LEC1: LEAFY COTYLEDON 1
LEC2: LEAFY COTYLEDON 2
NAA: 1-Naphthylacetic acid
PINs: PIN-FORMEDs
PKR2: PICKLE-RELATED 2
PLT2: PLETHORA2
PRC2: Polycomb Repressive Complex 2
*REV*: *REVOLUTA*
SAM: Shoot apical meristem
SERKs: SOMATIC EMBRYOGENESIS RECEPTOR-LIKE KINASE
SUS2: SUCROSE SYNTHASE 2
tZ: *trans*-zeatin
VAL1: VIVIPAROUS1/ABI3-LIKE 1
WOXs: WUSCHEL RELATED HOMEOBOXs
WUS: WUSCHEL
YUCs: YUCCAs
ZMP: Zeatin riboside-5' -monophosphate
