# Supplementary material for: Expression of AtLEC2 and AtIPTs promotes embryogenic callus formation and shoot regeneration in tobacco
Source: BMC Plant Biol. 2019 Jul 15;19:314. doi: 10.1186/s12870-019-1907-7 (PMC6633698; doi:10.1186/s12870-019-1907-7)
Supplement: Supplementary file 1 — Figure S1. Phenotype of seedlings grown on hormone free MS medium without DEX. Figure S2. Phenotype of mature plants grown in soil. Figure S3. PCR amplified the AtLEC2, AtIPT3, AtIPT7 and AtIPT9 fragments. Figure S4. Seedlings grown on 50 μM DEX condition. Figure S5. Seedlings grown on 20 μM DEX containing medium. Figure S6. Shoot regeneration from the callus. Table S1. Primers used in the study. (DOCX 1921 kb) [file 12870_2019_1907_MOESM1_ESM.docx]

**Supplementary Material**

**Expression of *AtLEC2* and *AtIPTs* Promotes Embryogenic Callus Formation and Shoot Regeneration in Tobacco**

Ke Li^1^, Juan Wang^3^, Chuanliang Liu^4^, Changsheng Li^2^, Jingjing Qiu^3^, Chuanzhi Zhao^2^，Han Xia^2^,Changle Ma^3^, Xingjun Wang^1 2 3^*, Pengcheng Li^2 3^*

* Correspondence: Corresponding Author: X.W. (email: xingjunw@hotmail.com) and P.L. (email: lpcsaas@outlook.com)


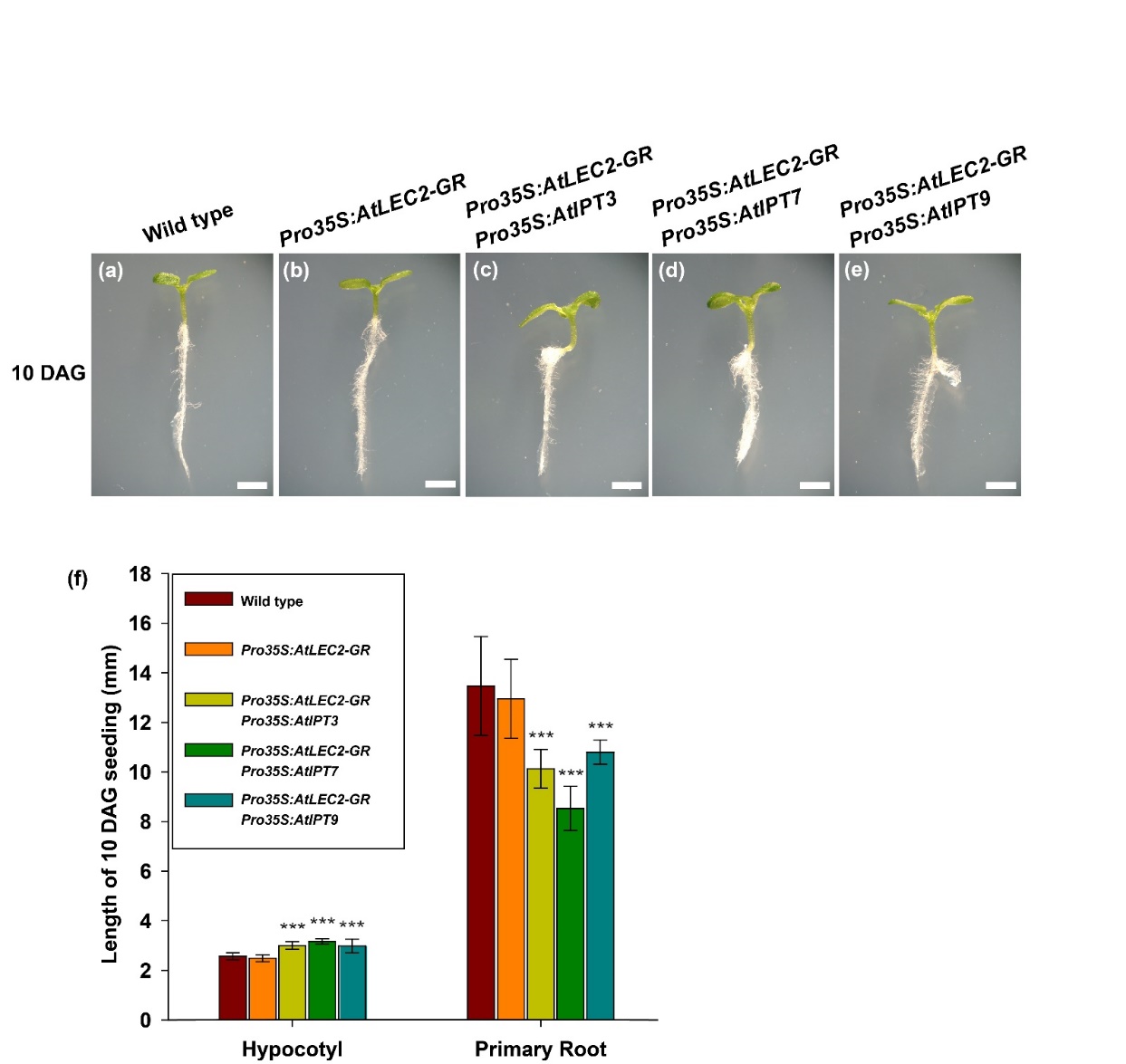


**Figure S1. Phenotype of seedlings grown on hormone free MS medium without DEX.** (a-e) 10 DAG seedlings of the indicated lines grown on hormone free MS medium without DEX. Scale bars = 3 mm. (f) The length of hypocotyl and primary root of the indicated lines. Values are mean ± standard errors form 20 replicates. Three asterisks indicate statistically significant differences (**^＊＊＊^***P*< 0.001, Student’s *t*-test) from the wild type.

**
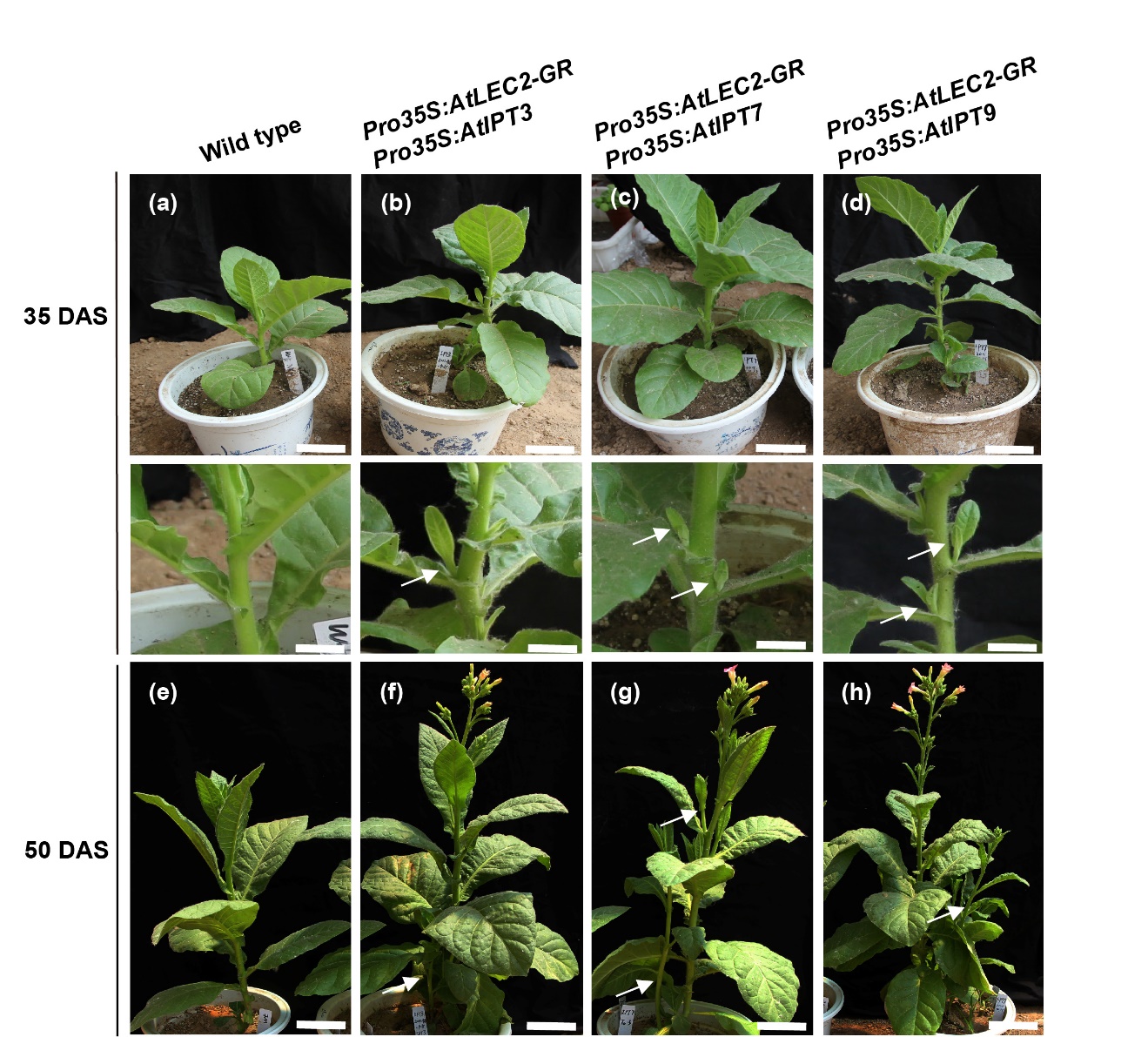
**

**Figure S2. Phenotype of mature plants grown in soil.** (a-d) The upper parts are the 35 DAS plants of indicated lines. Scale bars = 7.5 cm. White arrows point to axillary buds. The lower parts are the enlarged images. Scale bars = 2.5 cm. (e-h) 50 DAS plants of indicated lines. Scale bars = 8.5 cm. DAS, days after transformed in the soil; white arrows point to the lateral branches.

**
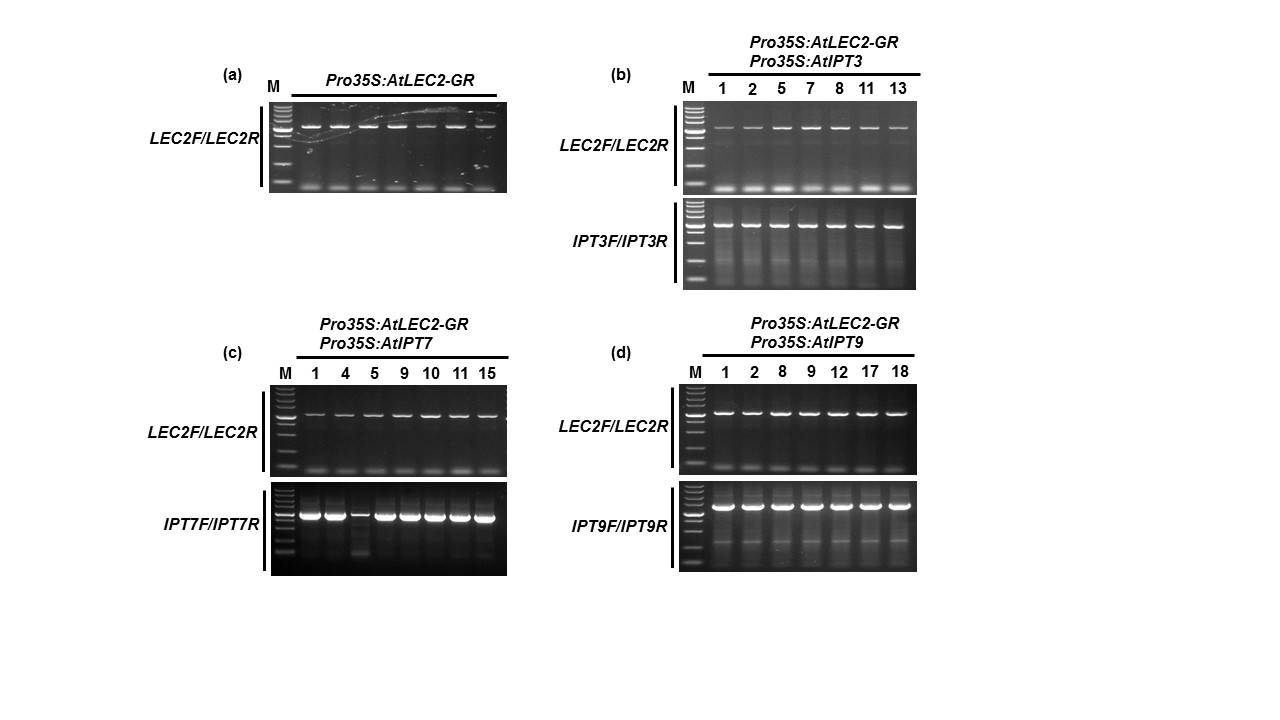
**

**Figure S3. PCR amplified the *AtLEC2*, *AtIPT3*, *AtIPT7* and** ***AtIPT9*** **fragments.** (a) Amplified *AtLEC2* fragment using specific primers in the indicated lines. (b-d) Amplified *AtLEC2*, *AtIPT3*, *AtIPT7* and *AtIPT9* fragments using specific primers in the indicated lines.

**
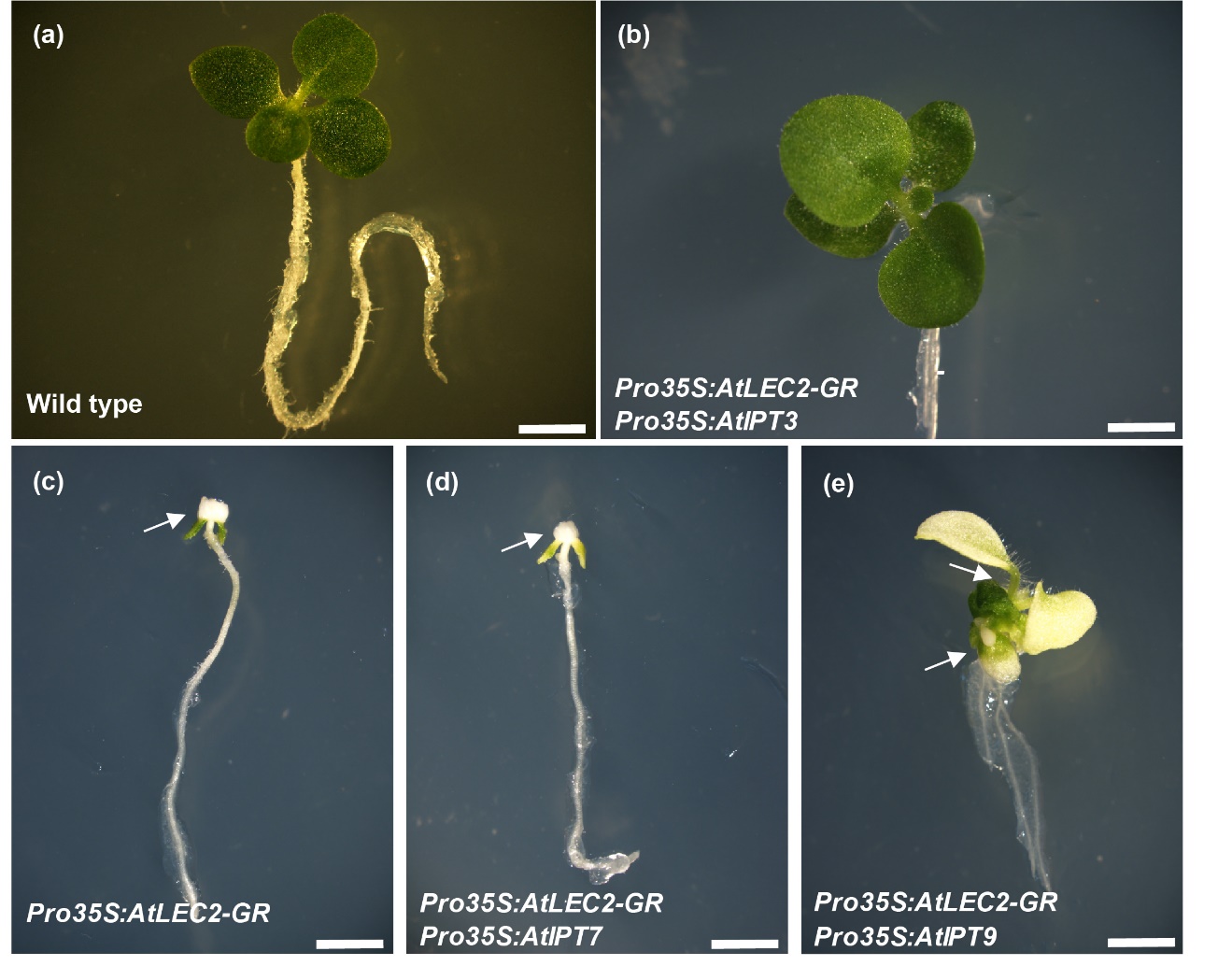
Figure S4. Seedlings grown on 50 μM DEX condition.** (a-e) Phenotype of 20 DAG seedlings of the indicated lines grown on 50 μM DEX condition*.* Scale bars = 3 mm. White arrows point to the embryonic callus.


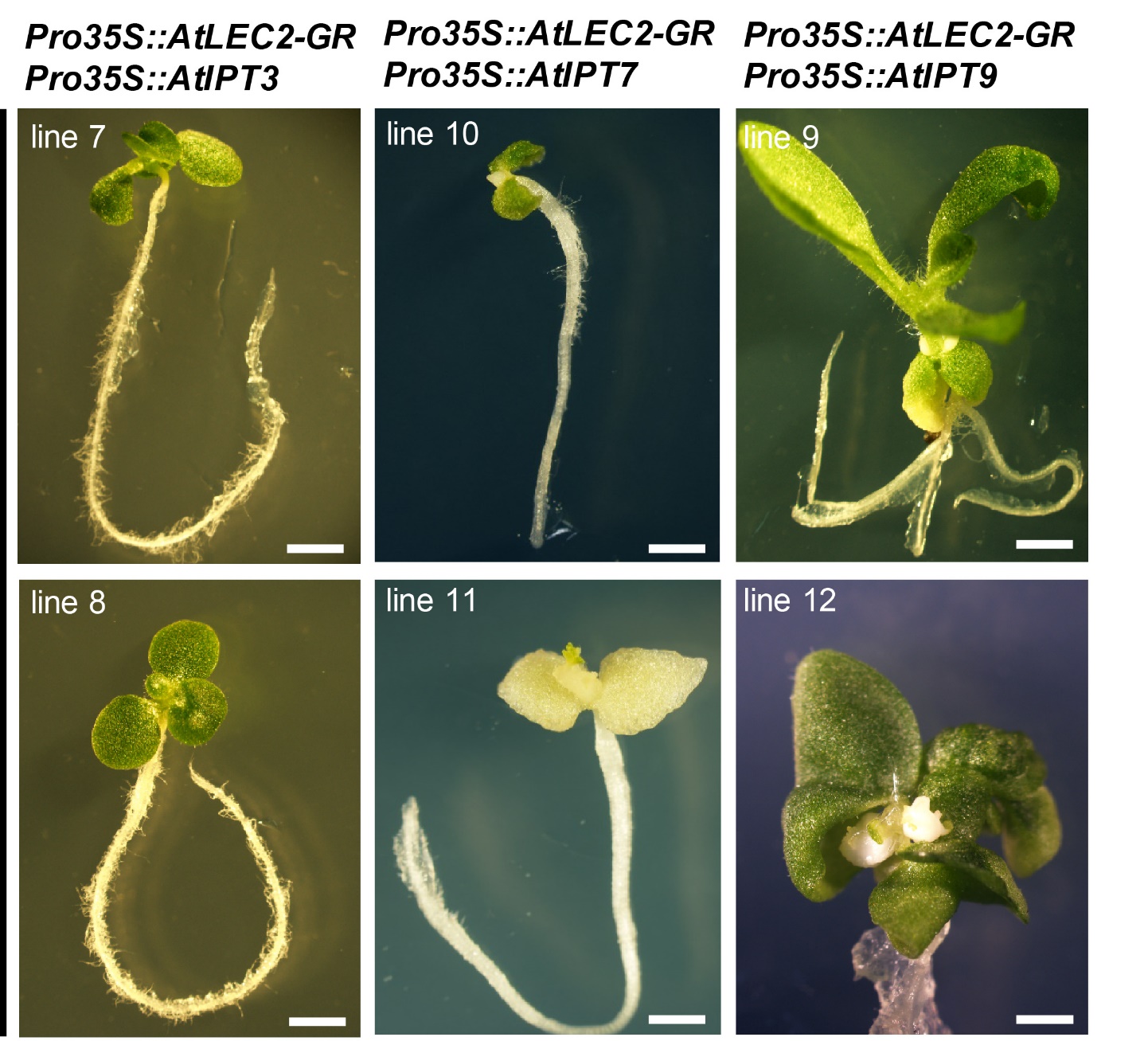
**Figure S5. Seedlings grown on** **20 μM DEX containing medium.** Two other representative transgenic lines of indicated transgenic seedlings were shown. Scale bars = 1.6 mm. lines idicated the line number of relevant *AtIPTs-OE AtLEC2-GR* transgeneic seedlings. The pictures were captured at 20 DAG.


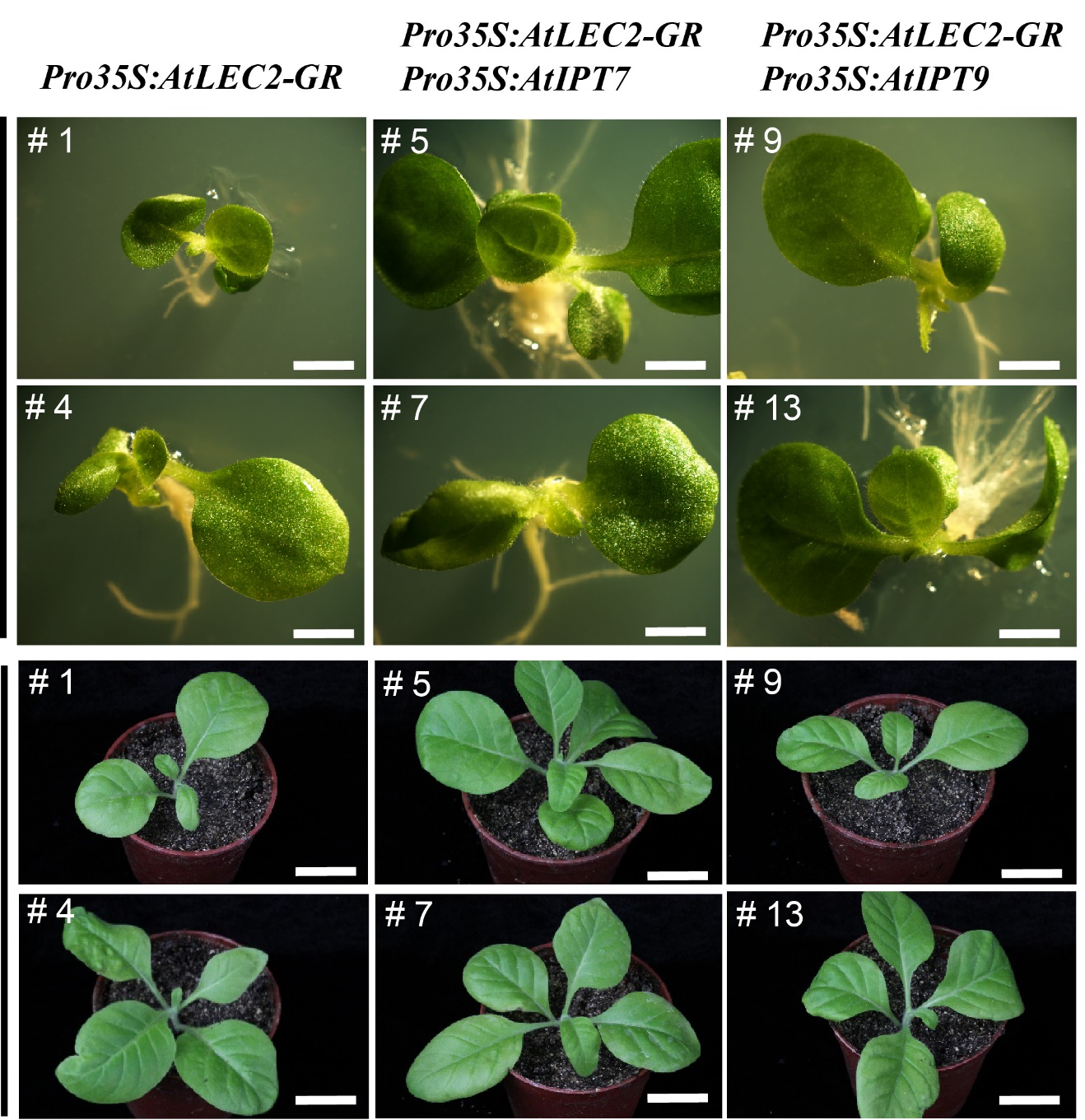
**Figure S6. Shoot regeneration from the callus.** Two representative regenerated shoots and seedlings of the indicated transgenic lines. Scale bars = 3 mm (shoots grown on MS medium). Scale bars = 3 cm (seedlings grown in soil).

**Table S1. Primers used in the study.**

| Purpose | Primer name | Sequence (5’-3’) |
| --- | --- | --- |
| Constructs | *AtIPT3F BamHI* | *GGATCCATGATCATGAAGATATCTATGGCT* |
|  | *AtIPT3R SalI* | GTCGACTCACGCCACTAGACACCGCGACAA |
|  | *AtIPT7F KpnI* | GGTACCATGAAGTTCTCAATCTCATCACTG |
|  | *AtIPT7R BamHI* | GGATCCTCATATCATATTGTGGGCTCTACT |
|  | *AtIPT9F BamHI*  *AtIPT9R* SalI | GGATCCATGGTGATTGGCAGTGGCGTATTT  GTCGACTTATGCTATTGCGCTTTCCACGCA |
